# Large-scale characterization of the bovine virome reveals viral diversity and community patterns in Northwest China

**DOI:** 10.3389/fmicb.2026.1924807

**Published:** 2026-09-22

**Authors:** Lingling Jiang, Yunyi Kong, Gang Zhang, Pu Wang, Min Fang, Qiang Liu, Sinong Zhang, Yong Li

**Affiliations:** School of Life Sciences, Ningxia University, Yinchuan, Ningxia, China

**Keywords:** beef cattle, phylogenetics, sampling season, viral co-detection, viral diversity, viral metagenomics, virome, virus isolation

## Abstract

Cattle play a critical role in global agriculture and food security while serving as hosts for diverse viruses with potential implications for animal and public health. To comprehensively characterize the bovine virome and its community patterns, we conducted a large-scale characterization of the beef cattle-associated virome in Northwest China using viral metagenomic sequencing. A total of 2,353 specimens, including nasal swabs, anal swabs, and serum, were collected from 794 cattle across 28 sites in Shaanxi, Gansu, and Ningxia, with nasal and anal swab specimens used for the metagenomic analyses. High-throughput sequencing identified viruses belonging to 14 phyla, 32 classes, 39 orders, 71 families, and 397 genera, revealing substantial viral diversity. Descriptive comparisons of the pooled libraries revealed differences in virome composition across geographic regions, farming systems, and sampling seasons, with the summer pool showing greater viral richness and contig abundance than the winter pool. Phylogenetic analyses revealed genetic relationships between several viral sequences detected in this study and viral lineages previously reported from humans and other animal hosts, raising hypotheses regarding broader host associations and potential cross-species transmission that require further investigation. Targeted PCR/RT-PCR screening and cell-culture-based virus characterization provided complementary validation of representative metagenomic findings and revealed frequent co-detection of multiple viral targets among the sampled cattle. Collectively, this study provides a large-scale characterization of the beef cattle-associated virome in Northwest China, expands current knowledge of viral diversity associated with beef cattle populations, and establishes a valuable resource for future virome surveillance, studies of viral evolution, and disease prevention.

## Introduction

Cattle are among the most important livestock species worldwide, providing essential sources of meat and contributing substantially to food security and sustainable agricultural development. In China, beef production has increased steadily in response to growing consumer demand, with annual beef consumption expected to reach approximately 10 million tons (Zhang and Hou, 2020). Currently, cattle production is still dominated by smallholder farming systems, although intensive and large-scale operations have expanded rapidly in some regions. Frequent movement of cattle between smallholder and commercial farms may facilitate the spread of infectious pathogens across herds and geographic regions, posing a persistent challenge to disease prevention and control (Alocilla and Monti, 2022). Viral diseases remain a major threat to the cattle industry, with both endemic and emerging viruses causing substantial economic losses through respiratory disease, enteric infections, reproductive disorders, and reduced productivity (Ambrose et al., 2023). Representative pathogens include bovine viral diarrhea virus (BVDV) (Xia et al., 2025), bovine coronavirus (BCoV) (Vlasova and Saif, 2021), bovine rotavirus (BRV) (Hou et al., 2025), and bovine astrovirus (BAstV) (Nelsen et al., 2023), all of which are widely distributed and have been associated with significant disease burdens in cattle populations.

Viral metagenomics, enabled by recent advances in high-throughput sequencing technologies, has transformed the detection and characterization of viral communities by allowing the unbiased identification of viral nucleic acids directly from clinical and environmental samples (Bassi et al., 2022). Compared with conventional diagnostic approaches, viral metagenomics provides high sensitivity for detecting both known and previously unrecognized viruses, making it a powerful tool for virus discovery, molecular epidemiology, and virome surveillance (Nooij et al., 2018). This approach has been successfully applied to characterize viral communities in a wide range of vertebrate and invertebrate hosts, including humans, bats, pigs, and poultry, as well as in environmental reservoirs, substantially expanding current understanding of viral diversity and evolution (Mokili et al., 2012). However, despite these advances, comprehensive large-scale studies simultaneously investigating both DNA and RNA viromes in beef cattle remain limited.

In the present study, we performed a large-scale characterization of the beef cattle virome in Northwest China using viral metagenomic sequencing. A total of 2,353 specimens, including nasal swabs, anal swabs, and serum, were collected from 794 cattle across 28 sampling sites in Shaanxi Province, Gansu Province, and the Ningxia Hui Autonomous Region. Only nasal and anal swab specimens were used for the metagenomic analyses reported in this study. We systematically characterized viral diversity and compared virome composition across geographic regions, farming systems, and sampling seasons. Furthermore, phylogenetic analyses were performed for representative DNA and RNA virus families, and targeted PCR/RT-PCR screening and cell-culture-based virus characterization were used to provide complementary validation of selected metagenomic findings. Collectively, this study provides a comprehensive overview of the bovine virome in Northwest China, expands current understanding of viral diversity and community patterns associated with beef cattle populations, and establishes a valuable resource for future virome surveillance, studies of viral evolution, and the prevention and control of bovine viral diseases.

## Methods

### Sample collection and processing

The sampling protocol was approved by the Animal Welfare and Ethics Committee of Ningxia University. All procedures were conducted in accordance with the Principles and Guidelines for Laboratory Animal Medicine issued by the Ministry of Science and Technology of China.

A total of 2,353 specimens were collected from 794 beef cattle at 28 sampling sites across Shaanxi, Gansu, and Ningxia, Northwest China (Figure 1). Detailed information on sampling dates, locations, sample numbers, and geographic coordinates is provided in Supplementary Table S1. Sampling was conducted between December 1, 2021, and June 20, 2023. Sampling sites were selected to capture variation in geographic location, farming system, and sampling season within Northwest China. At each site, cattle were enrolled using a blind, non-targeted sampling strategy without prior selection based on clinical signs, age, sex, or other demographic characteristics. The clinical health status of sampled cattle was assessed by trained veterinarians at the time of sampling based on observable clinical signs. Written informed consent was obtained from all farm owners before sample collection. Farm-level information was collected using a standardized questionnaire covering farming practices, vaccination history, and disease history; however, complete information was not consistently available across all sampling sites. The questionnaire template is provided in Supplementary Table S4.

**Figure 1:**
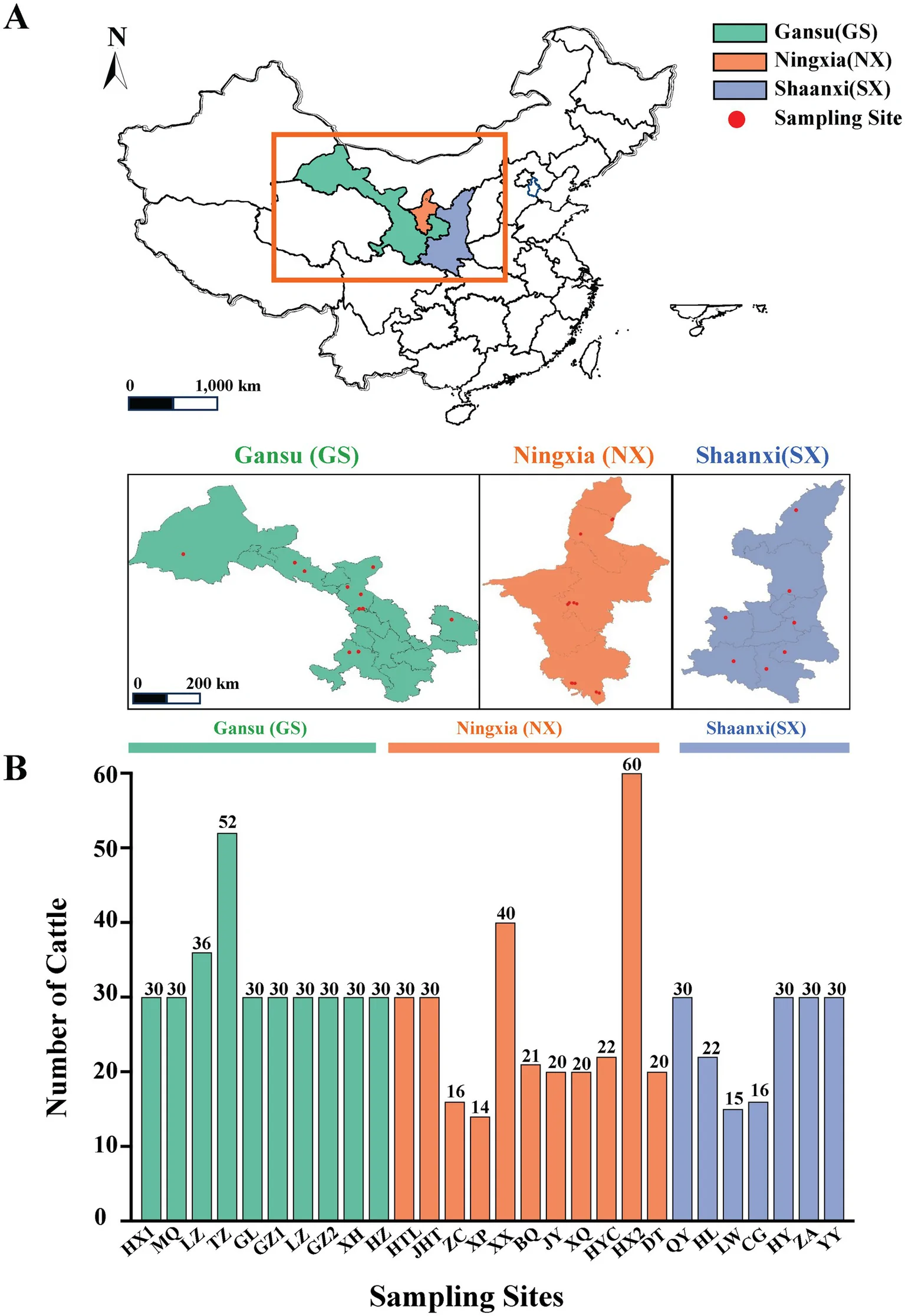
Geographic distribution of beef cattle sampling sites across Northwest China. **(A)** Geographic distribution of the 28 cattle sampling sites located in Gansu (GS), Ningxia Hui Autonomous Region (NX), and Shaanxi (SX). The inset map shows the location of the three regions within China. Red dots indicate individual sampling sites. **(B)** Number of beef cattle sampled at each collection site. Bars are grouped by province and correspond to the sampling locations shown in **(A)**. A total of 794 beef cattle were sampled from 28 sites, including 328 cattle from Gansu, 293 from Ningxia, and 173 from Shaanxi.

Each animal contributed up to three specimen types, including nasal swabs, anal swabs, and serum. Nasal swabs were collected by inserting sterile swabs into the nasal cavity and gently rotating them against the nasal mucosa. Anal swabs were obtained by inserting sterile swabs approximately 6–7 cm into the rectum and rotating them to collect mucosal material. Each swab was immediately placed into a sterile tube containing 3 mL of viral transport medium. Blood samples were collected from the tail vein, allowed to clot, and centrifuged to separate the serum. Serum samples were collected as part of the broader sampling framework but were not included in the metagenomic sequencing analyses reported in this study; only nasal and anal swab specimens were used for metagenomic library construction. All samples were transported to the laboratory in liquid nitrogen and stored at −80 °C until further processing. Sterile consumables were used throughout the sampling procedure to minimize the risk of cross-contamination.

### Sample preprocessing, pooling, and Illumina sequencing

Sample preprocessing and Illumina sequencing were performed according to the workflow shown in Figure 2. Nasal and anal swab suspensions were vortexed and centrifuged at 12,000 × *g* for 10 min at 4 °C to obtain clarified supernatants, which were sequentially filtered through 0.45-μm and 0.22-μm membrane filters (Millipore, MA, USA) to remove cellular debris. Only nasal and anal swab specimens were used for the metagenomic sequencing described in this study; serum samples collected during field sampling were not included in these metagenomic libraries.

**Figure 2:**
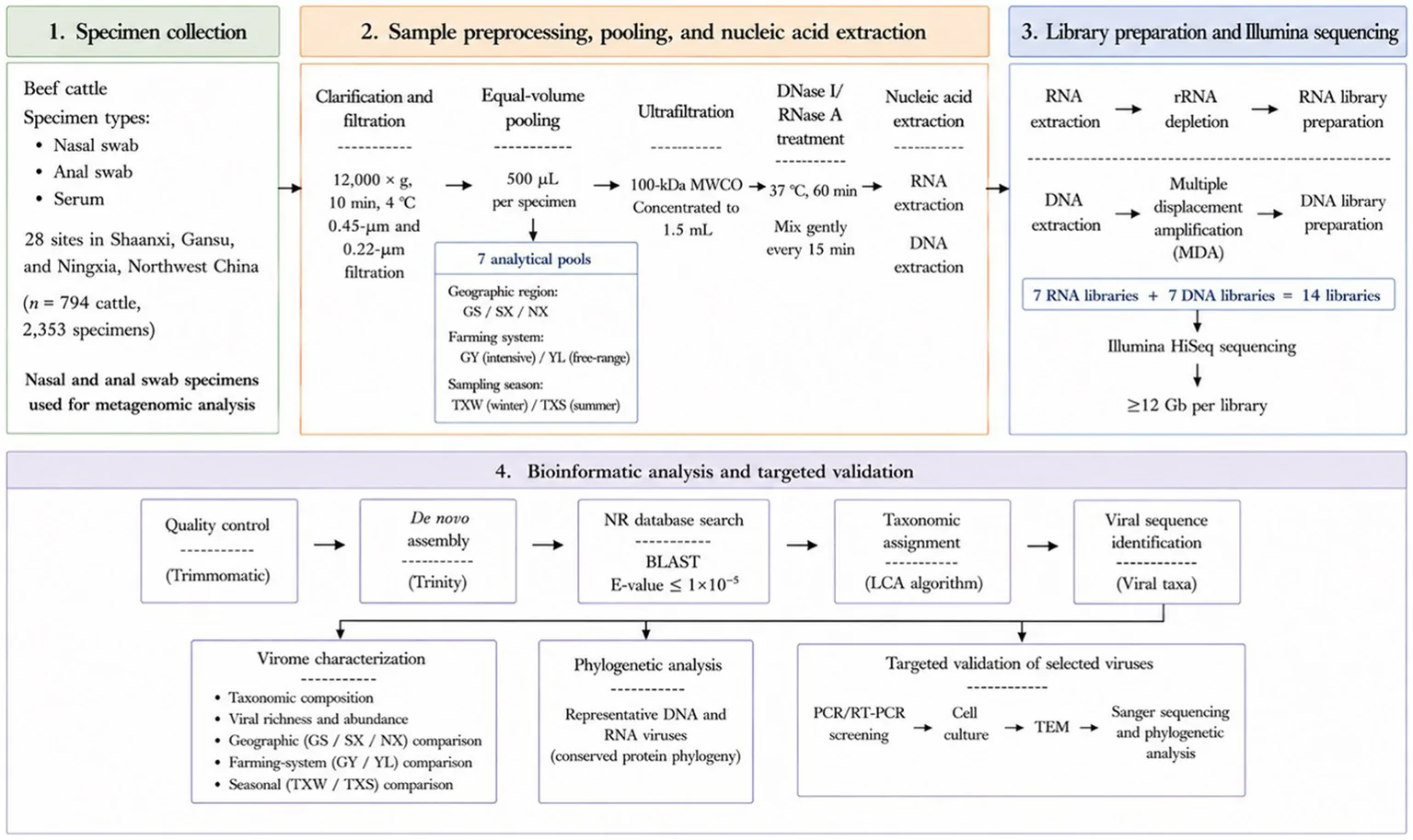
Workflow of viral metagenomic sequencing and bioinformatic analysis of the beef cattle virome. Overview of the experimental and bioinformatic workflow used for bovine virome profiling. Specimens were collected from beef cattle and processed for DNA/RNA extraction, metagenomic library preparation, and Illumina sequencing. Sequencing reads were subjected to quality control, host read removal, *de novo* assembly using Trinity, taxonomic annotation by NR/NT database searches and viral RefSeq confirmation, followed by taxonomic classification and open reading frame (ORF) prediction. Viral sequences were subsequently used for virome composition analyses, whereas complementary validation of selected metagenomic findings.

For metagenomic library construction, 500 μL of the filtered supernatant from each individual nasal or anal swab specimen was combined within the corresponding pooled group. Nasal and anal swab specimens were combined within each pool. Seven pooled groups were generated according to three predefined comparison schemes: geographic region (Gansu [GS], Shaanxi [SX], and Ningxia [NX]), farming system (intensive farming [GY] and free-range farming [YL]), and sampling season (winter [TXW] and summer [TXS]). These seven groups were not mutually exclusive; specimens included in the farming-system and seasonal comparisons were also represented within their corresponding geographic groups. In addition, the absence of independent biological replication at the pool level limits formal statistical inference regarding differences among groups. Thus, the three grouping schemes represented different classifications of the same overall sample collection rather than seven independent sample populations. Equal volumes of filtered supernatant were used for pooling, and pooling was performed before nucleic-acid extraction; therefore, nucleic-acid concentrations of individual specimens were not normalized before pool construction. The pooled specimens were subsequently concentrated to a final volume of 1.5 mL using 100-kDa molecular-weight-cutoff ultrafiltration devices (Millipore, MA, USA).

To reduce unprotected extracellular nucleic acids, each concentrate was treated with DNase I and RNase A (1 μL each; Takara, Dalian, China) at 37 °C for 60 min with gentle mixing every 15 min. Viral RNA was extracted using TRIzol reagent (Invitrogen, Thermo Fisher Scientific, USA), and seven RNA libraries were constructed using 2–4 μg of total RNA from each pool. Ribosomal RNA was removed using a commercial rRNA depletion kit (Vazyme Biotech, China). RNA libraries were prepared with the NEBNext® Ultra™ II RNA Library Prep Kit for Illumina (New England Biolabs, USA) according to the manufacturer’s instructions, including RNA fragmentation, first- and second-strand cDNA synthesis, end repair, adaptor ligation, and PCR amplification.

Total DNA was extracted using the TIANGEN Oral Swab DNA Extraction Kit (TIANGEN Biotech, China). Purified DNA was subjected to multiple displacement amplification (MDA) using the Illustra GenomiPhi V2 DNA Amplification Kit (GE Healthcare, USA). DNA libraries were subsequently prepared from 1 μg of amplified DNA using the NEBNext® Ultra™ DNA Library Prep Kit for Illumina (New England Biolabs, USA) following the manufacturer’s instructions.

The quality and concentration of all DNA and RNA libraries (14 libraries in total) were evaluated before sequencing. Paired-end sequencing was performed on the Illumina HiSeq platform, generating a minimum of 12 Gb of raw sequencing data for each library. The resulting sequencing data were subsequently subjected to the bioinformatic analyses described below, and selected metagenomic findings were further validated using targeted PCR/RT-PCR assays.

### Bioinformatic analysis of viral sequences

The bioinformatic workflow comprised quality control, *de novo* assembly, taxonomic assignment, and viral sequence identification (Figure 2). Raw sequencing reads were subjected to quality control to remove adapter-containing reads, reads containing excessive ambiguous bases, and low-quality reads. The resulting clean reads were used for downstream analyses, and sequencing quality was assessed based on Q20, Q30, GC content, and the proportion of clean reads.

Quality-filtered reads were assembled *de novo* using Trinity to generate contigs. Assembly quality was assessed using standard metrics, including the number and total length of contigs, maximum contig length, N50, N90, and GC content. The assembled contigs were compared against the NCBI non-redundant protein (NR) database using BLAST with an E-value threshold of ≤1 × 10^−5^. Taxonomic assignments were based on sequence-similarity matches to the NR database and were reported at the available taxonomic levels, including phylum, class, order, family, genus, and species. Contigs assigned to viral taxa were extracted for subsequent virome-specific analyses, whereas sequences without sufficient support for assignment to a specific taxonomic level were retained as unclassified or unassigned where appropriate. Contigs assigned to non-viral taxa, including eukaryotic, bacterial, and archaeal sequences, were excluded from downstream viral community analyses. Thus, host-derived sequences assigned to eukaryotic taxa were excluded at the post-assembly taxonomic filtering stage rather than through a separately documented pre-assembly host-genome mapping step.

Viral taxonomic profiles derived from the annotated sequences were used to characterize virome composition and taxonomic richness across the pooled metagenomic libraries. Sequences without sufficient support for assignment to a specific viral taxon were retained as unclassified where appropriate. Selected viral findings were subsequently examined by targeted molecular assays and phylogenetic analyses as described below.

For the comparative taxonomic analyses in Figures 3, 4, contig abundance was defined as the number of taxonomically assigned viral contigs associated with each taxon within a pooled library, whereas relative contig abundance represents the corresponding within-library proportion. Log10(x + 1) transformation was applied where indicated for visualization, while presence–absence analyses were based solely on taxon detection. Contig abundance was used as a descriptive sequence-based metric and was not interpreted as a direct measure of absolute viral abundance.

**Figure 3:**
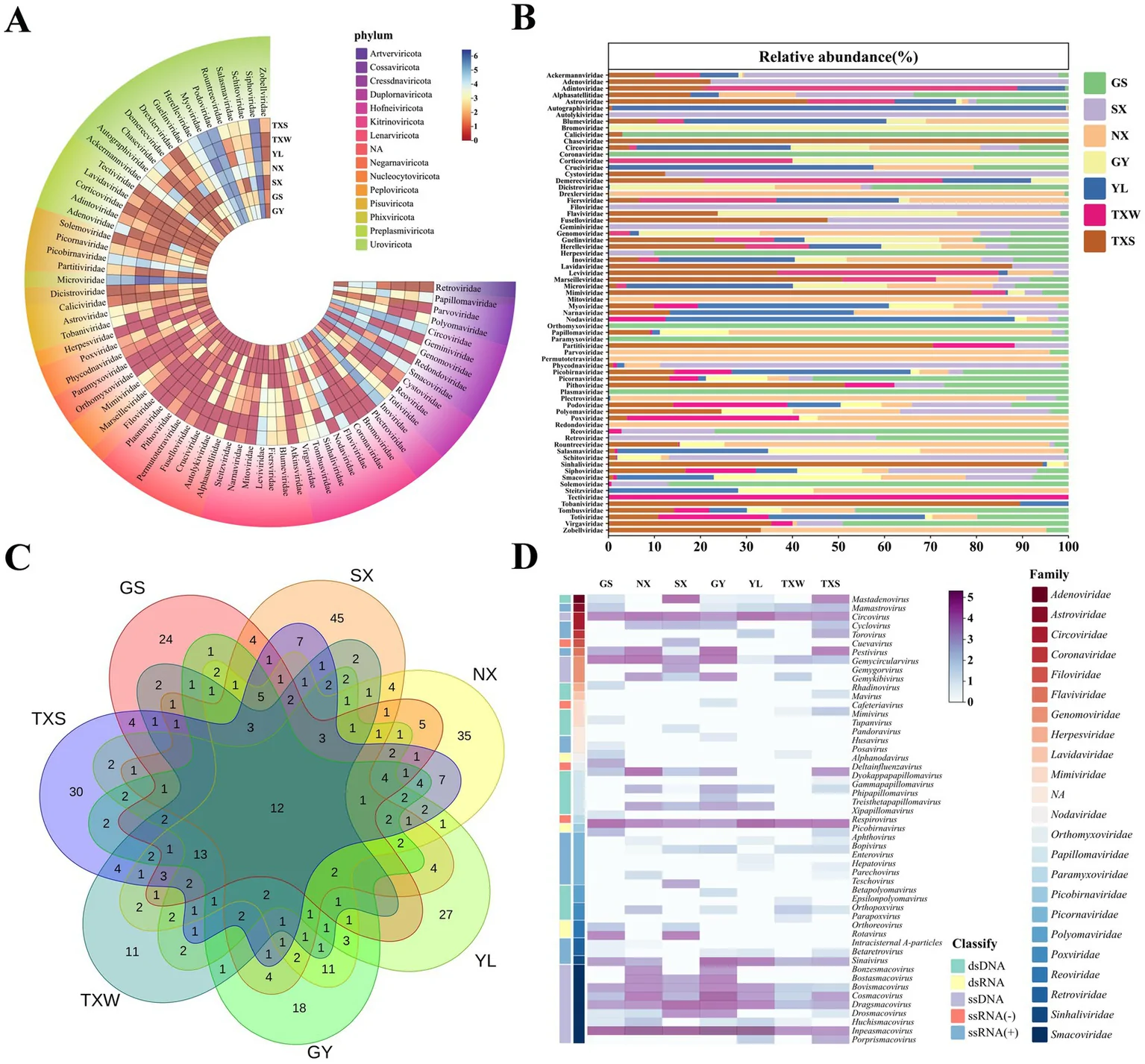
Composition and taxonomic patterns of the beef cattle virome across pooled groups. **(A)** Circular heatmap showing viral contig abundance at the family level across the seven pooled groups. Viral taxa are organized by phylum, and color intensity represents log_10_-transformed contig abundance [log_10_(x + 1)]. Numerical values are provided in Supplementary Table S10. **(B)** Stacked bar plot showing the relative abundance (%) of the dominant viral families in each pooled group. Colored segments represent individual viral families, and each bar is normalized to 100%. Numerical values are provided in Supplementary Table S11. **(C)** Overlap of viral genera among the seven pooled groups based on presence–absence analysis. Numbers in each region represent the number of viral genera shared by the corresponding combination of pooled groups or unique to an individual group. Numerical values are provided in Supplementary Table S12. **(D)** Heatmap showing viral contig abundance of animal-associated viral genera across the seven pooled groups. Rows represent viral genera and columns represent pooled groups. Color intensity indicates log_10_-transformed contig abundance [log_10_(x + 1)]. Viral genome types and taxonomic affiliations are indicated alongside the heatmap. Numerical values are provided in Supplementary Table S13. GS, Gansu; SX, Shaanxi; NX, Ningxia Hui Autonomous Region; GY, intensive farming (Guyuan); YL, free-range farming (Yulin); TXW, winter; TXS, summer.

**Figure 4:**
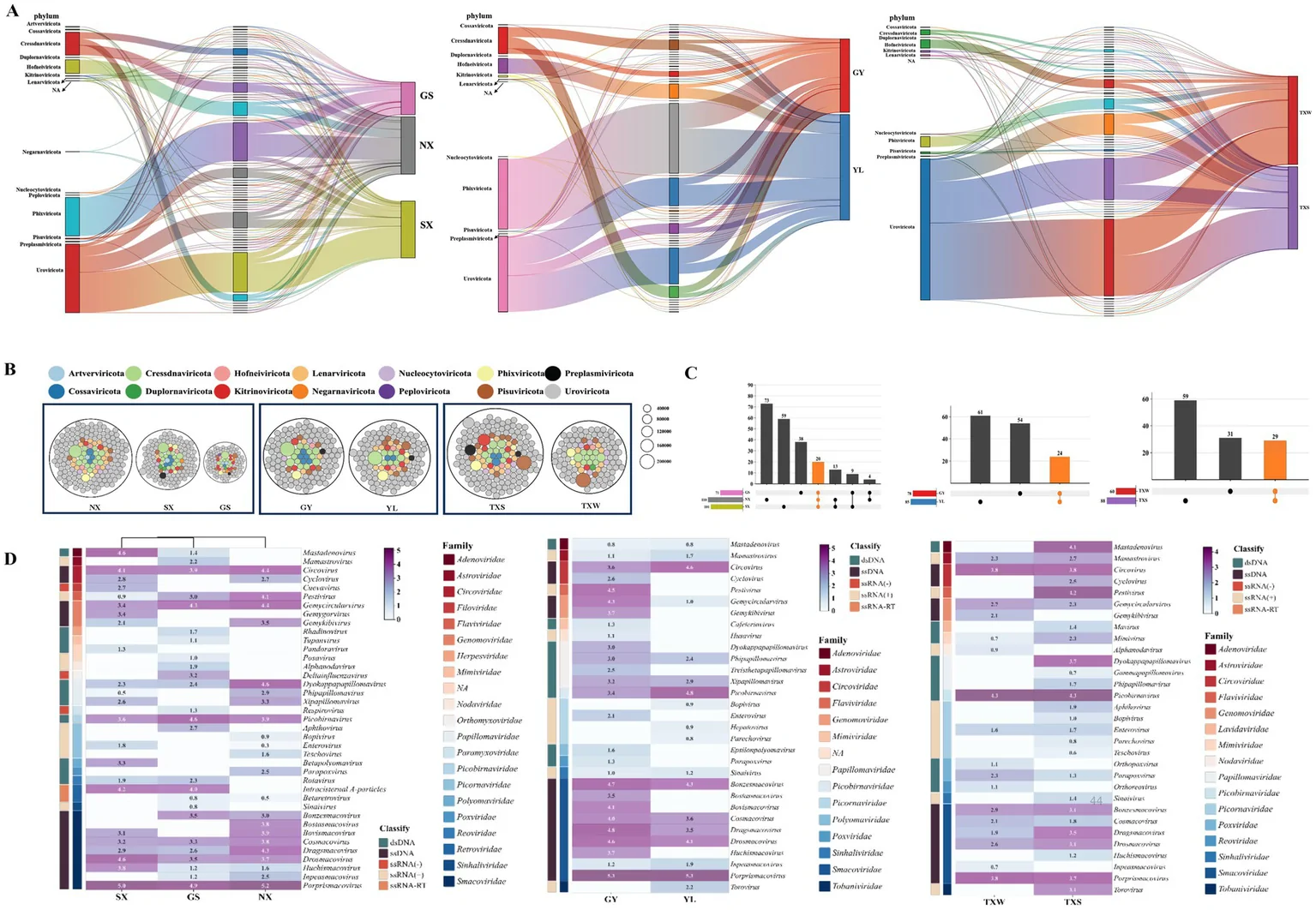
Comparison of bovine virome composition across geographic regions, farming systems, and seasons. Panels are organized according to the ecological factor being compared: Left, geographic regions (GS, SX, and NX); Middle, farming systems (GY, intensive farming; YL, free-range farming); and Right, seasons (*TX*W, winter; TXS, summer). **(A)** Sankey diagrams showing the distribution of viral contig abundance from phylum to family across pooled groups. The width of each flow is proportional to contig abundance. Numerical data are provided in Supplementary Table S14. **(B)** Circle packing plots illustrating the hierarchical organization of viral taxa from phylum to genus. Circle size is proportional to viral contig abundance, and nested circles represent taxonomic hierarchy. Numerical data are provided in Supplementary Table S15. **(C)** UpSet plots showing the numbers of shared and unique viral genera among pooled groups within each ecological factor. Vertical bars indicate the size of each intersection, whereas connected dots identify the corresponding group combinations. **(D)** Heatmaps showing viral contig abundance of animal-associated viral genera across pooled groups. Rows represent viral genera and columns represent pooled groups. Color intensity indicates log_10_-transformed contig abundance [log_10_(x + 1)]. Colored annotations indicate viral genome types and taxonomic affiliations. Unless otherwise indicated, abundance values shown in panels **(A,B,D)** represent contig abundance and should be interpreted as sequence-based descriptive measures rather than estimates of absolute viral abundance. GS, Gansu; SX, Shaanxi; NX, Ningxia Hui Autonomous Region; GY, intensive farming (Guyuan); YL, free-range farming (Yulin); TXW, winter; TXS, summer.

### Phylogenetic analysis of bovine-associated viruses

Phylogenetic analyses were performed using representative viral sequences identified in this study. Reference sequences were retrieved from the NCBI GenBank database based on sequence similarity to the viral sequences identified in this study. Depending on the viral taxon and the purpose of each phylogenetic analysis, representative reference sequences were selected to include relevant host-associated lineages, geographic origins (regions or countries), and/or recognized viral subtypes, where appropriate, to provide phylogenetic context. Multiple sequence alignments were generated using MEGA version 7.0. Phylogenetic relationships were inferred using either amino acid sequences of conserved viral proteins or nucleotide sequences of representative genomic regions, depending on the characteristics of each viral taxon. Maximum likelihood (ML) phylogenetic trees were constructed using MEGA, with branch support assessed by 1,000 bootstrap replicates. The optimal nucleotide or amino acid substitution model was selected according to the lowest Bayesian Information Criterion (BIC). Alignment positions containing gaps or missing data were treated using partial deletion with a site coverage cutoff of 95%; thus, positions containing more than 5% gaps or missing data were excluded from phylogenetic reconstruction.

For highly divergent viral sequences, phylogenetic analyses were additionally performed using amino acid sequence alignments to improve alignment accuracy. Phylogenetic trees were visualized and edited using FigTree (version 1.4.4). Viral genome annotation and sequence visualization were performed using GATU and SnapGene, respectively.

### Validation of viral detection and virus isolation

To provide targeted validation of selected metagenomic findings, a subset of 293 cattle from 10 of the 28 sampled farms was selected from the 794 cattle included in the field-sampling cohort. These animals were part of the same sampling cohort used to generate the corresponding pooled metagenomic libraries and were selected based on the availability of sufficient residual nasal and anal swab material for individual molecular testing. Targeted PCR/RT-PCR screening was performed for six bovine viruses: bovine viral diarrhea virus (BVDV), bovine coronavirus (BCoV), bovine rotavirus (BRV), bovine norovirus (BNoV), bovine astrovirus (BAstV), and bovine herpesvirus 1 (BoHV-1). These six viruses were prioritized because they were detected in the metagenomic dataset and represent epidemiologically important bovine viruses associated with respiratory, enteric, and systemic diseases. Their selection also encompassed both RNA and DNA viruses, thereby providing targeted validation of selected findings representing different viral genome types detected by metagenomic sequencing. For each animal, equal volumes of nasal and anal swab suspensions were combined for nucleic-acid extraction and viral detection. Viral nucleic acids were extracted using the TianGen DNA/RNA Extraction Kit (DP315; TianGen Biotech, China) according to the manufacturer’s instructions. Viral RNA was reverse transcribed into complementary DNA (cDNA) using the PrimeScript™ II First Strand cDNA Synthesis Kit (Takara, Japan). PCR and RT-PCR assays were performed using virus-specific primers designed in this study based on conserved genomic regions (Supplementary Table S5). Amplification products were analyzed by agarose gel electrophoresis, and positive amplicons were purified and confirmed by Sanger sequencing.

Selected PCR/RT-PCR-positive samples were subsequently subjected to virus isolation. Clarified swab supernatants were inoculated onto confluent Madin–Darby bovine kidney (MDBK) cell monolayers and passaged through four successive blind passages. Cytopathic effects (CPE) were monitored daily by light microscopy. Culture supernatants collected from each passage were examined by PCR or RT-PCR to assess the presence of the corresponding viral nucleic acids. Representative culture-positive samples were further characterized by transmission electron microscopy (TEM) to visualize viral particles. Selected culture-positive samples were subsequently confirmed by Sanger sequencing and phylogenetic analysis. Target genomic regions included the 5′ untranslated region (5′ UTR) of BVDV, the ORF1a region of BAstV, and the glycoprotein B (*gB*) gene of BoHV-1. For selected BVDV isolates, near-complete viral genomes were obtained by combining PCR amplification with next-generation sequencing (NGS). Whenever possible, samples positive for a single targeted virus were preferentially selected for virus isolation to minimize potential co-detected viruses and facilitate downstream virological characterization.

## Results

### Construction and quality assessment of the viral dataset

From December 1, 2021, to June 20, 2023, a total of 794 beef cattle were sampled at 28 sites across Shaanxi, Gansu, and the Ningxia Hui Autonomous Region in Northwest China, including 173 cattle from Shaanxi, 328 from Gansu, and 293 from Ningxia (Figure 1A). A total of 2,353 specimens, including nasal swabs, anal swabs, and serum, were collected. Only nasal and anal swab specimens were included in the metagenomic sequencing analyses reported in this study. The number of cattle sampled at each site is shown in Figure 1B, and detailed sampling information, including collection dates, locations, geographic coordinates, sample types, and sample numbers, is provided in Supplementary Table S1.

To compare virome composition across geographic regions, farming systems, and sampling seasons, the same sample cohort was classified into seven pooled groups: GS (Gansu), SX (Shaanxi), and NX (Ningxia Hui Autonomous Region) for geographic regions; GY (intensive farming, Guyuan) and YL (free-range farming, Yulin) for farming systems; and TXW (winter) and TXS (summer) for seasonal comparisons (Table 1). These seven groups were not mutually exclusive; specimens represented in the farming-system and seasonal groups were also included within their corresponding geographic groups. Based on these grouping schemes, 14 metagenomic libraries, comprising seven DNA libraries and seven RNA libraries, were constructed (Table 2).

**Table 1:** Experimental grouping of beef cattle samples for pooled metagenomic library construction.

Group	Grouping factor	Sampling sites (*n*)	Cattle (*n*)	Description
GS	Geographic region	12	328	Gansu Province, China
SX	Geographic region	6	173	Shaanxi Province, China
NX	Geographic region	8	293	Ningxia Hui Autonomous Region, China
YL	Farming system	2	30	Free-range, Yulin City, China
GY	Farming system	5	71	Intensive farming, Guyuan City, China
TXW	Season	2	60	Winter, Tongxin County, China
TXS	Season	2	80	Summer, Tongxin County, China

**Table 2:** Summary of metagenomic sequencing and assembly statistics for the bovine virome.

Library	Raw reads	Clean reads, *n* (%)	Assembled contigs	Annotated contigs, n (%)	Viral contigs
GS-DNA	72,065,342	72,065,302(100.00%)	450,448	220,112(48.87%)	105,005
GS-RNA	71,359,076	71,359,068(100.00%)	206,419	53,291(25.82%)	1,913
SX-DNA	81,169,020	81,168,986(100.00%)	659,535	254,723(38.62%)	103,089
SX-RNA	72,038,762	72,038,760(100.00%)	208,894	55,874(26.75%)	571
NX-DNA	72,595,948	71,920,224(99.07%)	471,347	224,351(47.60%)	104,863
NX-RNA	43,878,126	43,640,480(99.46%)	106,869	40,034(37.46%)	1,647
YL-DNA	77,464,894	76,842,678(99.20%)	385,874	188,799(48.93%)	84,622
YL-RNA	66,507,388	65,748,730(98.86%)	97,523	33,309(34.16%)	1,518
GY-DNA	74,613,166	73,937,314(99.09%)	425,967	190,247(44.66%)	102,521
GY-RNA	41,899,140	41,693,986(99.51%)	142,954	47,610(33.30%)	659
TXW-DNA	41,698,250	41,424,816(99.34%)	399,916	177,046(44.27%)	96,633
TXW-RNA	43,022,414	42,730,502(99.32%)	150,688	49,851(33.08%)	1,277
TXS-DNA	43,166,158	42,999,984(99.62%)	451,158	212,502(47.10%)	114,012
TXS-RNA	46,012,542	45,898,062(99.75%)	207,418	67,213(32.40%)	1,953
Total	847,490,226	843,468,892(99.53%)	4,365,010	1,814,962 (41.58%)	720,283

Illumina sequencing generated a total of 847,490,226 raw reads, comprising 462,772,778 DNA reads and 384,717,448 RNA reads. After quality filtering, 843,468,892 clean reads were retained, corresponding to an overall retention rate of 99.53%. The proportion of clean reads exceeded 98% across all libraries, with the GS and SX DNA and RNA libraries showing the highest read-retention rates (Figure 5A). DNA libraries generally yielded more sequencing reads than RNA libraries, with the SX group producing the largest number of reads.

**Figure 5:**
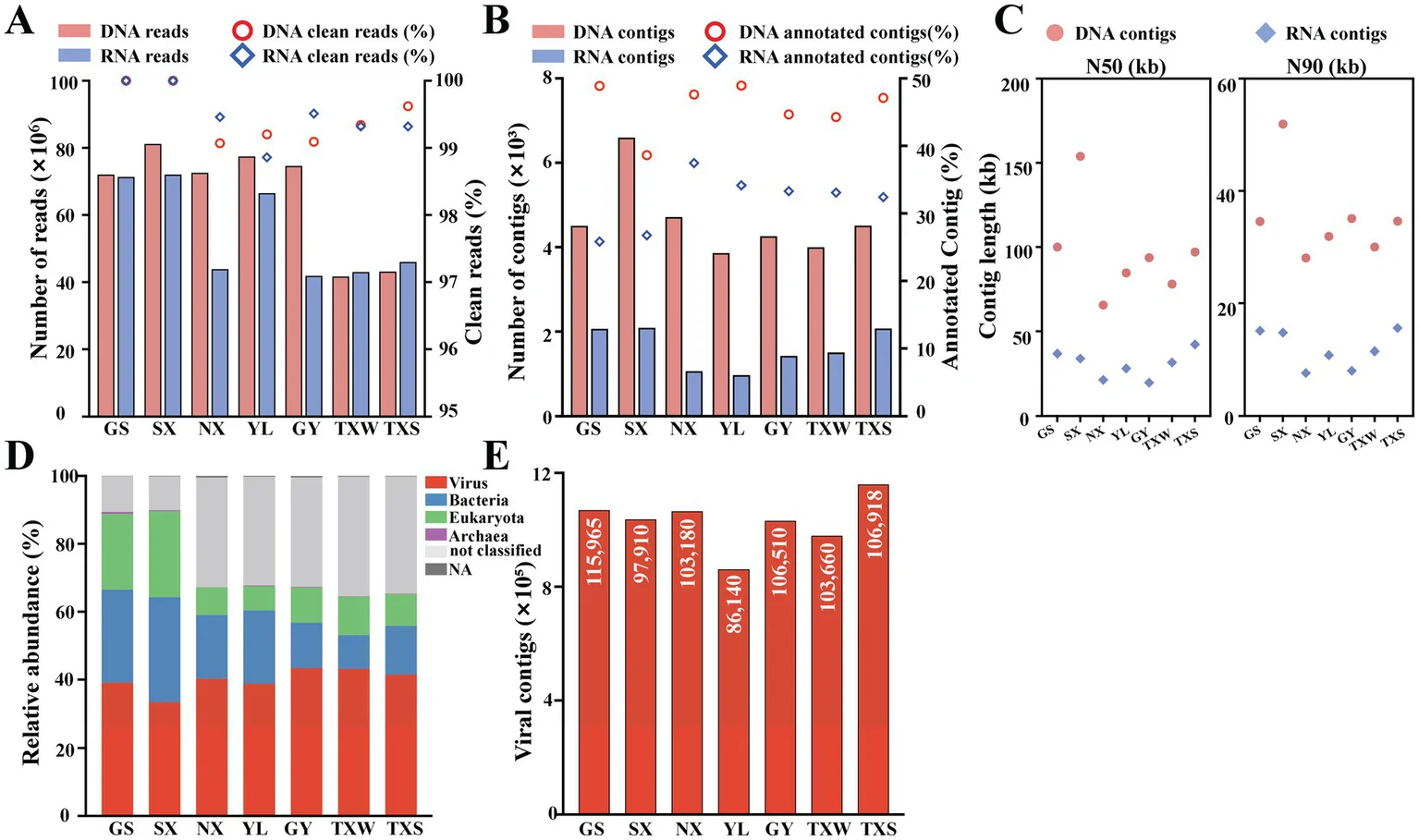
Sequencing output, assembly quality, and taxonomic annotation of the bovine virome dataset. **(A)** Numbers of raw reads and clean reads generated from DNA and RNA metagenomic libraries across the seven pooled groups. Open symbols indicate the percentage of clean reads after quality filtering. Detailed sequencing statistics are provided in Supplementary Table S6. **(B)** Numbers of assembled contigs and taxonomically annotated contigs obtained from DNA and RNA libraries. Open symbols indicate the proportion of annotated contigs. Detailed assembly statistics are provided in Supplementary Table S7. **(C)** Assembly quality of DNA and RNA metagenomic libraries based on N50 and N90 values. Numerical values are provided in Supplementary Table S8. **(D)** Relative taxonomic composition of assembled contigs across the seven pooled groups, including viral, bacterial, eukaryotic, archaeal, unclassified, and unassigned (NA) sequences. Detailed taxonomic annotation data are provided in Supplementary Table S9. **(E)** Numbers of viral contigs identified in each pooled group following taxonomic annotation. GS, Gansu; SX, Shaanxi; NX, Ningxia Hui Autonomous Region; GY, intensive farming (Guyuan); YL, free-range farming (Yulin); TXW, winter; TXS, summer.

Quality-filtered reads were assembled *de novo* using Trinity, generating 4,409,674 contigs. Assembly characteristics were assessed based on contig length distributions and N50 and N90 statistics across the libraries (Figure 5C). Following sequence processing and taxonomic annotation, 4,365,010 contigs, comprising 3,244,245 DNA-derived and 1,120,765 RNA-derived contigs, were retained for subsequent analyses. Of these, 1,814,962 contigs (41.58%) were included in the taxonomic classification output, comprising viral, bacterial, eukaryotic, archaeal, unclassified, and unassigned sequences. DNA-derived contigs accounted for the majority of the assembled sequences, whereas RNA-derived contigs represented more than 60% of the taxonomically annotated contigs (Figure 5B).

Taxonomic classification identified 720,283 viral contigs, 363,223 bacterial contigs, 253,298 eukaryotic contigs, and 4,316 archaeal contigs, together with 469,404 unclassified contigs and 4,438 sequences without taxonomic assignments (Table 2). Viral contigs constituted a substantial component of the taxonomically classified sequences across the pooled libraries, with more than 10^5^ viral contigs assigned to viral taxa in the GS, SX, NX, GY, and TXS groups (Figures 5D,E). Collectively, these results indicate substantial sequencing depth and broad taxonomic representation across the metagenomic libraries, providing a basis for subsequent characterization of bovine-associated viral diversity and comparative virome patterns.

### Viral community variation across geographic regions, farming systems, and seasons

Based on read counts, Uroviricota showed the highest sequence representation at the phylum level, accounting for 13,851,531 reads (49.10%), whereas Astroviridae showed the highest sequence representation at the family level, with 7,832,806 reads (27.76%). At the contig level, taxonomic composition across the seven pooled libraries was visualized using raw contig counts after log10(x + 1) transformation (Figure 3A), while the proportional distribution of dominant viral families was further summarized using relative contig abundance (Figure 3B). Taxonomic richness varied among pooled groups, with the SX and GS groups exhibiting the highest phylum-level richness (14 phyla each), while the SX group showed the greatest family richness (48 families). Despite marked variation in community composition, 12 viral genera—*Circovirus, Cosmacovirus, Dragsmacovirus, Drosmacovirus, Porprismacovirus, Gemycircularvirus, Microvirus, Picobirnavirus, Nitunavirus, Cequinquevirus, Saphexavirus,* and *Sextaecvirus*—were detected across all pooled groups, representing a shared component of the viral community detected in cattle-derived specimens (Figure 3C and Supplementary Table S2). The overall virome comprised bacteriophages as well as viruses associated with vertebrate and other eukaryotic hosts. Because detection of viral sequences in bovine-derived specimens does not necessarily indicate infection of cattle, animal-associated viral taxa were further examined separately from the overall viral community. In total, 52 animal-associated viral genera representing diverse genome types (ssRNA, dsRNA, ssDNA, and dsDNA) were identified. Their distribution across the pooled libraries showed substantial variation in contig abundance, with Smacoviridae showing the highest contig abundance at the family level and *Porprismacovirus* at the genus level (Figure 3D).

Viral community composition was further compared across geographic regions, farming systems, and sampling seasons (Figure 4). Among the geographic groups, the NX group exhibited a higher overall viral contig abundance than the SX and GS groups, whereas the latter two displayed relatively similar community compositions (Figure 4A). The hierarchical distribution of viral taxa from phylum to genus also varied among the pooled groups within the geographic, farming-system, and seasonal comparisons (Figure 4B). Presence–absence analysis revealed both shared and group-specific viral genera within each comparison (Figure 4C). Nevertheless, 20 viral genera were shared among all three regions, indicating a substantial shared component of the virome across the sampled regions, including multiple animal-associated viral taxa. Comparisons between farming systems revealed broadly similar viral richness in the intensive (GY) and free-range (YL) groups, with both containing 11 phyla and 40 families and with 78 and 85 genera detected, respectively. The summer group (TXS) exhibited greater viral richness than the winter group (TXW) at the phylum (13 vs. 11), family (46 vs. 37), and genus (88 vs. 60) levels, together with a higher overall viral contig abundance. The distribution and contig abundance of animal-associated viral genera also varied among the pooled groups (Figure 4D). These observations describe differences among the pooled libraries included in the present dataset. However, because each comparison was based on pooled libraries without independent biological replication, within-group variability could not be estimated and the independent effects of geography, farming system, and sampling season could not be formally assessed. Accordingly, these patterns should be interpreted as descriptive group-level observations rather than evidence that these factors independently shaped the bovine-associated viral community.

### Phylogenetic analysis of bovine-associated viruses

Phylogenetic analyses were performed using representative viral sequences identified in the metagenomic dataset based on conserved viral proteins or genomic regions (Figures 6, 7). A total of 14 vertebrate-associated viral families, including six DNA virus families and eight RNA virus families, were represented. Most viral sequences clustered with previously reported vertebrate-associated viruses, whereas several sequences formed distinct phylogenetic branches, indicating genetic divergence from their closest reference sequences.

**Figure 6:**
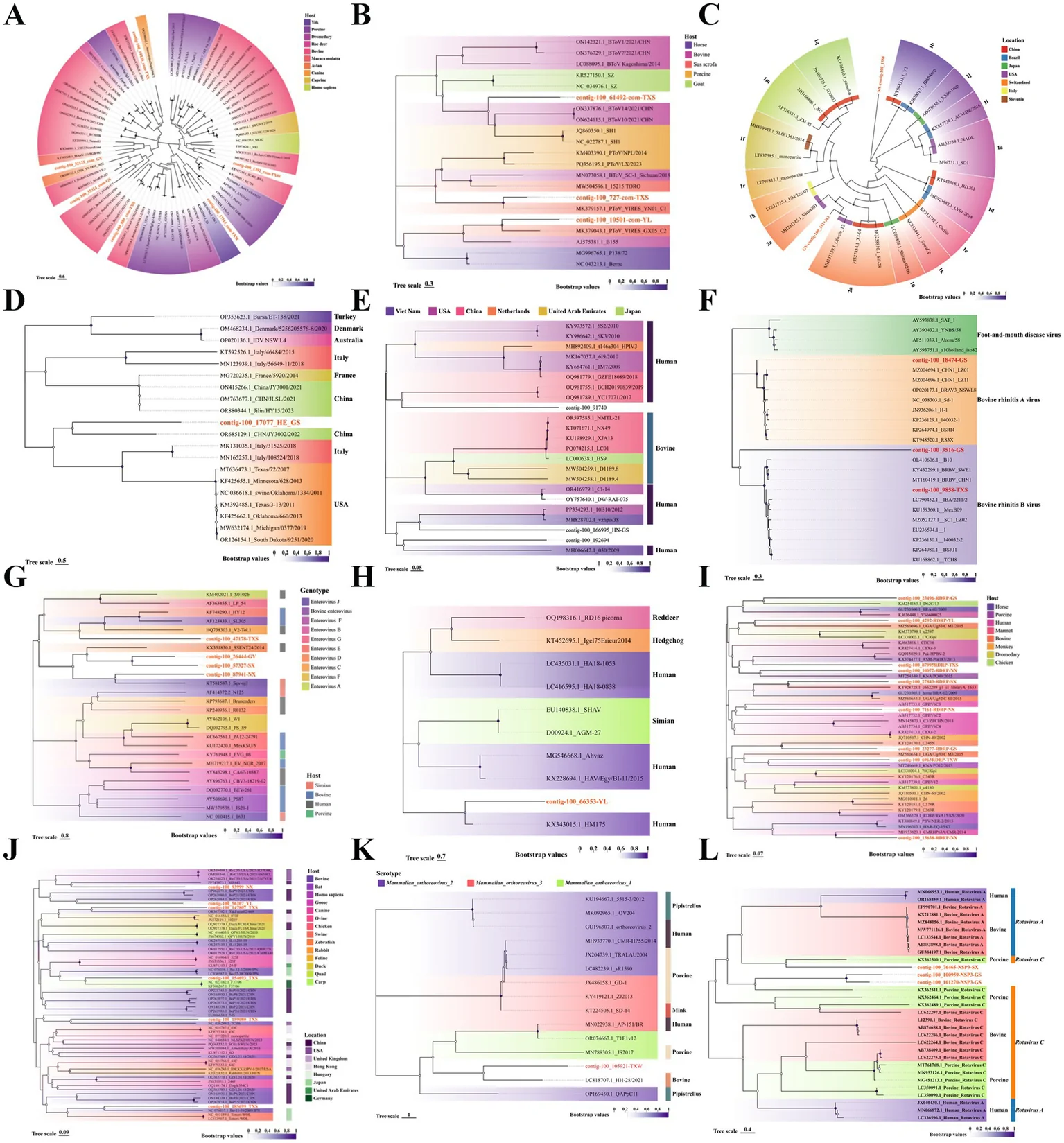
Phylogenetic analysis of representative DNA viruses identified in beef cattle. Maximum-likelihood phylogenetic trees of representative DNA virus families detected in this study, including **(A)** Adenoviridae, represented by two near-complete atadenovirus genomes; **(B)** *Circoviridae*, represented by nine circovirus genomes; **(C)** *Genomoviridae*, represented by genomes belonging to the *Gemycircularvirus* and *Genomovirus* lineages; **(D)** *Herpesviridae*, represented by a gammaherpesvirus genomic fragment; **(E)** *Papillomaviridae*, represented by 16 papillomavirus genomes; and **(F)** *Polyomaviridae*, represented by one betapolyomavirus genome. Viral sequences identified in this study are highlighted in red. Branch support was assessed using 1,000 bootstrap replicates.

**Figure 7:**
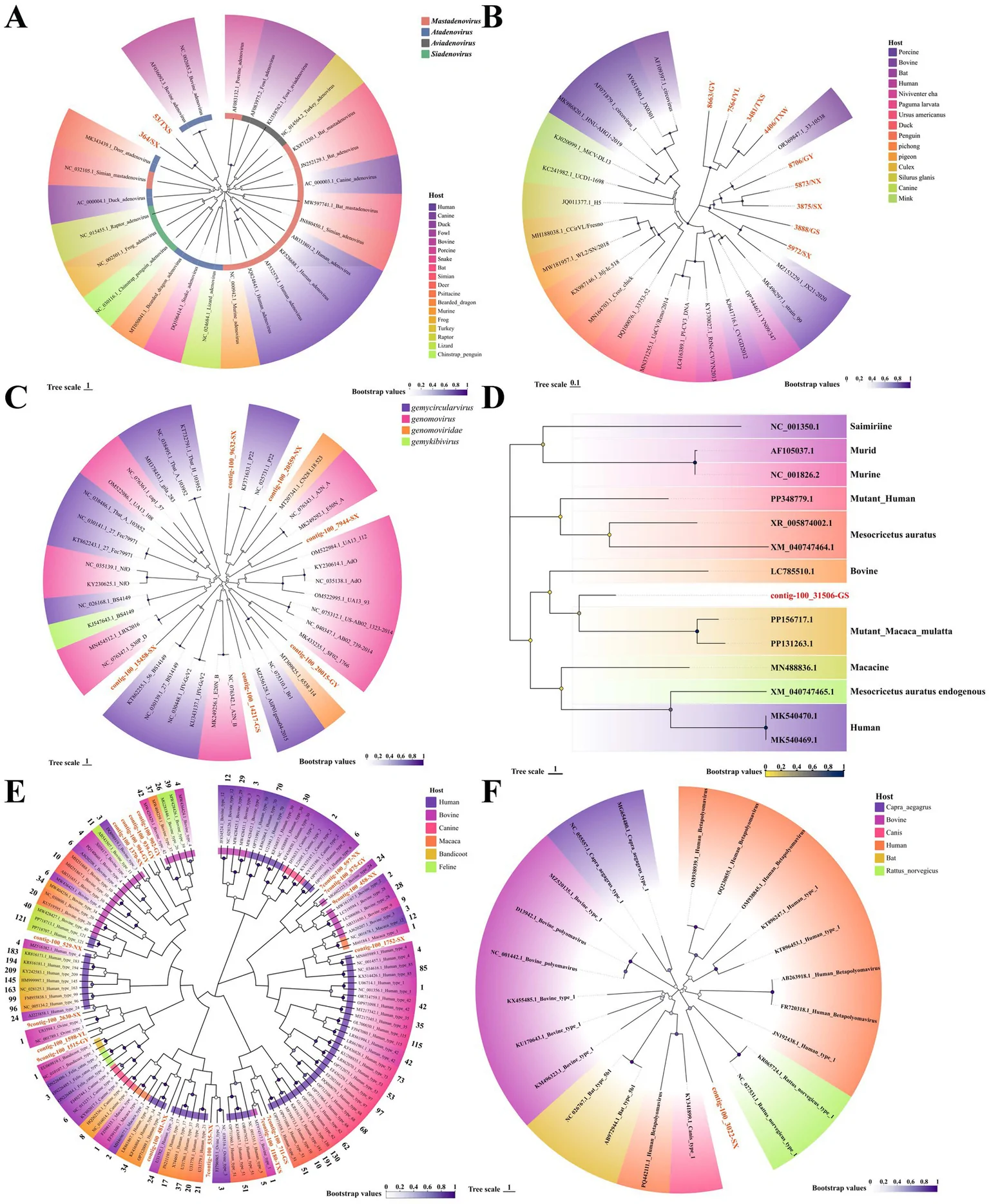
Phylogenetic analysis of representative RNA viruses identified in beef cattle. Maximum-likelihood phylogenetic trees of representative RNA viruses detected in this study, including **(A)** astroviruses (*Astroviridae*), **(B)** toroviruses (*Coronaviridae*), **(C)** bovine viral diarrhea viruses (*Flaviviridae*), **(D)** influenza D virus (*Orthomyxoviridae*), **(E)** bovine respirovirus 3 (*Paramyxoviridae*), **(F)** picobirnaviruses (*Picobirnaviridae*), **(G)** aphthoviruses, **(H)** enteroviruses, **(I)** hepatoviruses, **(J)** other representative picornaviruses (*Picornaviridae*), and **(K,L)** orthoreoviruses and rotaviruses (*Reoviridae*). Trees were constructed using complete or near-complete viral genomes or representative conserved genomic regions, depending on the viral taxon. Viral sequences identified in this study are highlighted in red. Branch support was assessed using 1,000 bootstrap replicates.

DNA viruses exhibited substantial phylogenetic diversity across multiple virus families (Figure 6). Two near-complete atadenovirus genomes shared high nucleotide identities (98.6–99.3%) with reference sequences and clustered within the bovine Atadenovirus serotype 4 lineage (Figure 6A). Nine circovirus genomes included both highly conserved sequences and more divergent lineages, with nucleotide identities ranging from 86.33 to 100% relative to their closest reference sequences (Figure 6B). Sequences belonging to the Genomoviridae clustered within the Gemycircularvirus and Genomovirus lineages (Figure 6C), whereas a gammaherpesvirus fragment grouped with reference sequences previously reported from bovine and primate hosts (Figure 6D). Sixteen papillomavirus genomes predominantly clustered with bovine papillomaviruses, while several sequences showed phylogenetic proximity to papillomavirus reference sequences associated with humans (Figure 6E). In addition, one betapolyomavirus genome exhibited the highest sequence similarity to a rodent-associated polyomavirus reference sequence (Figure 6F). Collectively, these results demonstrate that the DNA viral sequences identified in this study included both well-characterized bovine viruses and genetically related viruses with diverse host associations.

RNA viruses also displayed extensive phylogenetic diversity (Figure 7). Most astrovirus sequences clustered with bovine astroviruses, whereas several were closely related to viruses previously identified in dogs, non-human primates, and yaks (Figure 7A). Torovirus sequences were distributed among both bovine- and porcine-associated lineages (Figure 7B). Metagenomic BVDV sequences were classified into the BVDV-2a and BVDV-1b subtypes (Figure 7C). An influenza D virus sequence showed high nucleotide identity (99.2%) with previously reported strains (Figure 7D), whereas bovine respirovirus 3 sequences clustered with isolates reported from China and Southeast Asia (Figure 7E). Picobirnavirus and picornavirus sequences were distributed across multiple host-associated lineages. Notably, four enterovirus-related sequences were phylogenetically related to *Enterovirus J* and *Enterovirus D* (Figure 7H), while representative picornaviruses clustered with bovine rhinitis viruses and hepatitis A-like viruses (Figures 7F–J). Members of the Reoviridae included both orthoreoviruses and rotaviruses, several of which clustered with reference sequences previously reported from human and porcine hosts (Figures 7K,L). Overall, these phylogenetic analyses revealed extensive genetic diversity and phylogenetic relationships with viral lineages previously reported from diverse vertebrate hosts.

### PCR detection and co-detection patterns

To provide complementary validation of selected metagenomic findings, a total of 293 cattle from 10 farms were screened by PCR or RT-PCR for six selected bovine viruses, including bovine viral diarrhea virus (BVDV), bovine coronavirus (BCoV), bovine rotavirus (BRV), bovine norovirus (BNoV), bovine astrovirus (BAstV), and bovine herpesvirus 1 (BoHV-1). Agarose gel electrophoresis confirmed successful amplification of all target viruses, with amplicon sizes ranging from 286 to 652 bp (Figure 8A).

**Figure 8:**
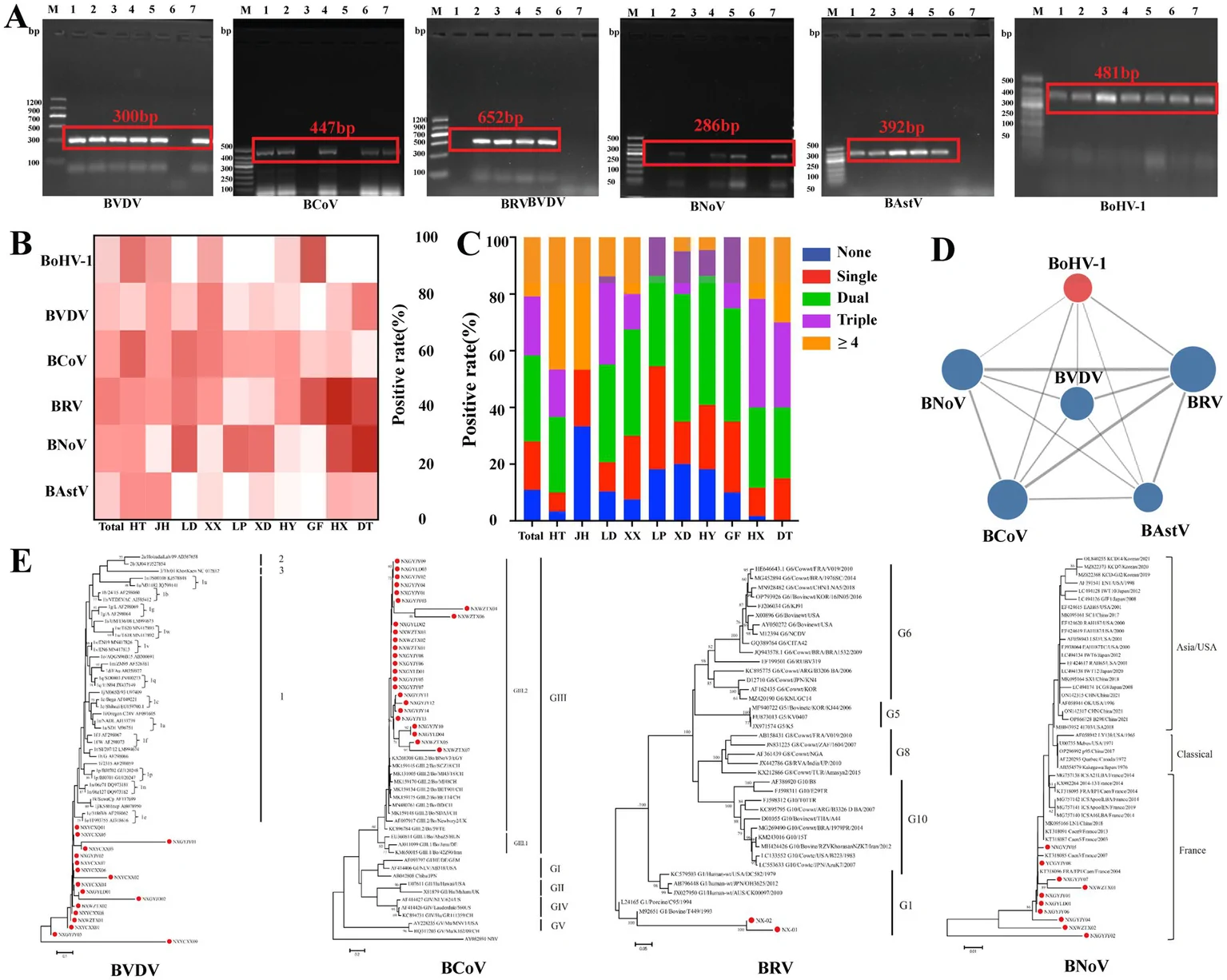
PCR detection, co-detection patterns, and phylogenetic characterization of bovine viruses. **(A)** Agarose gel electrophoresis showing PCR/RT-PCR amplification products of six bovine viruses, including bovine viral diarrhea virus (BVDV, 300 bp), bovine norovirus (BNoV, 447 bp), bovine coronavirus (BCoV, 652 bp), bovine rotavirus (BRV, 286 bp), bovine astrovirus (BAstV, 392 bp), and bovine herpesvirus 1 (BoHV-1, 481 bp). **(B)** Heatmap showing the detection rate (%) of the six bovine viruses across the ten sampled farms. Numerical values are provided in Supplementary Table S16. **(C)** Distribution of virus-negative, single-virus, dual-virus, triple-virus, and ≥4-virus detection across the sampled farms. Numerical values are provided in Supplementary Table S17. **(D)** Network analysis of viral co-detection patterns. Node size is proportional to virus detection rate, and edge thickness represents the frequency of co-detection between virus pairs. Numerical values are provided in Supplementary Table S18. **(E)** Maximum-likelihood phylogenetic trees of representative viral sequences obtained by targeted PCR/Sanger sequencing, including BVDV (5′ UTR), BNoV (RdRp), BRV (VP7), and BCoV (N gene). Viral sequences obtained in this study are highlighted in red. Branch support was assessed using 1,000 bootstrap replicates.

Overall, at least one of the six viral targets was detected in 89.08% of the cattle, whereas no target was detected in 10.92% (Figure 8B). Among the six viruses, BRV showed the highest detection rate (59.39%), followed by BNoV (48.12%) and BCoV (45.73%), whereas BVDV (32.08%), BAstV (25.26%), and BoHV-1 (23.89%) were detected at comparatively lower frequencies. The proportion of cattle positive for at least one target virus varied among farms, ranging from 66.67 to 100%, with different viruses predominating at individual sites, including BCoV at HT, BNoV at LD, LP, XD, and DT, and BRV at HX.

Multiple viral targets were detected in 71.92% of the cattle. Single-virus detection accounted for 17.06%, whereas dual-virus detection, triple-virus detection, and detection of four or more viruses accounted for 30.38, 20.82, and 20.82% of cattle, respectively (Figure 8C). High-order co-detection (≥4 viruses) was most frequently observed in the HT (46.67%), JH (46.67%), and DT (30.00%) farms. The most common co-detection combinations were BCoV + BRV + BNoV and BRV + BNoV. Co-detection network analysis showed that BCoV, BRV, and BNoV had the highest connectivity within the network of the six targeted viruses (Figure 8D).

Representative PCR amplicons were confirmed by Sanger sequencing. Phylogenetic analyses showed that BVDV 5′ UTR sequences obtained by targeted PCR/Sanger sequencing (OQ513857–OQ513873) belonged to the BVDV-1e subtype, BNoV RdRp sequences were classified as genotype GIII.2, BRV VP7 sequences belonged to genotype G1, and BCoV nucleocapsid (N) gene sequences clustered with previously reported BCoV reference sequences (Figure 8E).

### Virus isolation and characterization

PCR/RT-PCR-positive bovine swab samples were subjected to virus isolation using MDBK cell monolayers. Representative viruses, including bovine viral diarrhea virus (BVDV), bovine astrovirus (BAstV), and bovine herpesvirus 1 (BoHV-1), were selected for further characterization (Figure 9). A summary of virus isolation, post-culture PCR/RT-PCR positivity, and sequencing results is presented in Table 3, and detailed sample-level information is provided in Supplementary Table S3.

**Figure 9:**
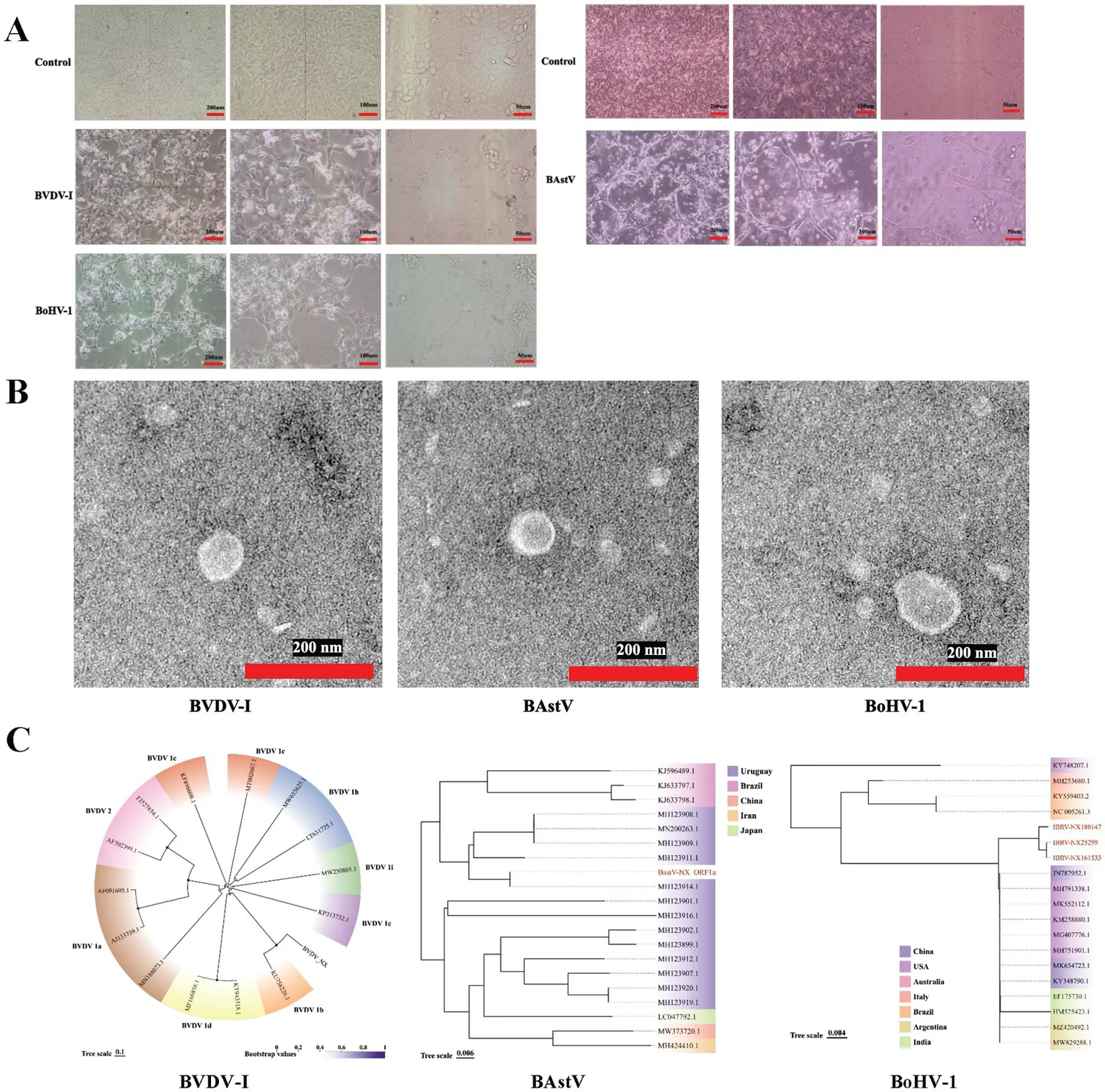
Isolation and characterization of representative bovine viruses. **(A)** Cytopathic effects (CPE) observed in MDBK cells during cell-culture isolation of representative bovine viruses, including bovine viral diarrhea virus (BVDV-1), bovine astrovirus (BAstV), and bovine herpesvirus 1 (BoHV-1). Representative images from sequential virus passages are shown. **(B)** Transmission electron microscopy (TEM) images of viral particles observed in representative culture-positive MDBK cell samples. Viral morphology is consistent with enveloped BVDV-1 and BoHV-1, and non-enveloped BAstV. Scale bars = 200 nm. **(C)** Maximum-likelihood phylogenetic analyses of representative viral sequences based on the BVDV 5′ untranslated region (5′ UTR), BAstV ORF1a, and BoHV-1 glycoprotein B (gB) gene sequences. Viral sequences obtained in this study are highlighted in red. Branch support was assessed using 1,000 bootstrap replicates.

**Table 3:** Summary of virus isolation, post-culture PCR detection, and sequencing outcomes for representative bovine viruses.

Virus	PCR/RT-PCR-positive samples (*n*)	Post-culture PCR/RT-PCR-positive samples (*n*)	Sequenced isolates (*n*)	Isolation rate (%)
BVDV-1	52	20	8	15.38
BAstV	37	23	1	2.70
BoHV-1	52	39	3	5.77

For BVDV, 52 RT-PCR-positive samples were subjected to cell culture, of which 20 remained RT-PCR-positive after cell culture. Eight viral isolates were obtained, including one for which a near-complete genome was generated and classified as subtype BVDV-1d. Infected MDBK cells exhibited characteristic cytopathic effects (CPE), including cell aggregation, detachment, and shrinkage (Figure 9A). Transmission electron microscopy (TEM) revealed enveloped viral particles approximately 50–60 nm in diameter (Figure 9B). Phylogenetic analysis of the near-complete genome further supported classification of this isolate within the BVDV-1d lineage (Figure 9C).

For BAstV, 37 RT-PCR-positive samples were subjected to cell culture, of which 23 remained RT-PCR-positive after cell culture, and one viral sequence was obtained for further characterization. Infected MDBK cells exhibited cell aggregation and plaque-like structures (Figure 9A). TEM revealed non-enveloped viral particles approximately 28–35 nm in diameter with star-like morphological features (Figure 9B). Phylogenetic analysis based on the ORF1a region showed 100% nucleotide identity with a previously reported BAstV reference sequence (Figure 9C).

For BoHV-1, 52 PCR-positive samples were subjected to cell culture, of which 39 remained PCR-positive after cell culture, and three viral sequences were obtained for further characterization. Infected MDBK cells exhibited pronounced CPE, including cell atrophy and increased intercellular spacing (Figure 9A). TEM revealed enveloped viral particles approximately 170 nm in diameter (Figure 9B). Phylogenetic analysis based on the glycoprotein B (*gB*) gene showed that the characterized sequences clustered with previously reported BoHV-1 reference sequences from the United States and China (Figure 9C).

Collectively, the cell-culture, PCR/RT-PCR, TEM, and sequence-based analyses provided complementary validation of selected viral findings identified by metagenomic sequencing.

## Discussion

Viral metagenomics has become a powerful approach for comprehensively characterizing viral communities in clinical and environmental samples, enabling the rapid and unbiased identification of both known and previously unrecognized viruses (Shi et al., 2018) (Li et al., 2015). Using this approach, we found that the viral community detected in beef cattle-derived specimens exhibited broad taxonomic richness, comprising 71 viral families and 397 viral genera, including 22 families and 52 genera associated with animal hosts. Compared with previous virome studies in sheep (Zhang et al., 2024), pigs (Nantel-Fortier et al., 2022), dairy cattle (Yan and Yu, 2024), and horses (Park and Kim, 2020), the present study identified broad taxonomic richness in the beef cattle virome. However, direct comparisons among studies should be interpreted cautiously because reported virome richness and diversity can be influenced by differences in host populations, specimen types, sampling intensity, sequencing depth, sample processing, and bioinformatic pipelines. Accordingly, the relatively high taxonomic richness observed in this study may reflect a combination of biological variation and methodological differences among studies.

An important consideration in interpreting this diversity is that the viral community detected in cattle-derived specimens includes viruses associated with different host groups. A substantial component of the overall virome consisted of bacteriophages, whereas a subset of the detected viruses was associated with vertebrate or other eukaryotic hosts. Accordingly, the term “bovine virome” is used here to describe the viral community detected in specimens collected from cattle rather than to imply that all detected viruses directly infect cattle. Detection of viral nucleic acids in bovine specimens alone does not establish host tropism, active infection, pathogenicity, or risk to animal health. Therefore, animal-associated viral taxa were examined separately where appropriate, and their biological significance should be interpreted cautiously.

Comparative analyses revealed differences in bovine virome composition across geographic regions, farming systems, and sampling seasons. The summer group exhibited greater viral richness and contig abundance than the winter group. Seasonal differences in environmental temperature, vector activity, animal management, and environmental exposure could potentially contribute to these patterns (Li et al., 2015) (Roper et al., 2025). However, these variables were not directly measured in the present study. Moreover, although the summer and winter groups were both sampled in Tongxin County, they were collected during different sampling periods and may have differed in farm composition, management conditions, environmental exposures, and other unmeasured variables. Therefore, the observed differences cannot be independently attributed to season. Similarly, because the intensive and free-range groups were sampled from different locations, geographic differences could not be disentangled from farming-system differences. These comparisons may also have been influenced by potential confounding factors, including cattle age, breed composition, diet, vaccination history, antimicrobial use, baseline health status, and other farm-level characteristics that were not uniformly available across all sampling sites. Importantly, because each comparison was based on pooled libraries without independent biological replication, within-group variability could not be estimated and formal inference regarding the independent effects of season, geography, or farming system was not possible. Accordingly, the observed differences should be regarded as descriptive group-level associations rather than evidence that these ecological factors independently shaped the bovine-associated viral community. Despite the observed variation among groups, 12 viral genera were detected across all pooled groups, indicating a shared component of the viral community detected across the sampled cattle populations.

The pooling strategy should also be considered when interpreting the comparative virome analyses. Equal volumes (500 μL) of individual swab suspensions were combined within each analytical pool before concentration and nucleic-acid extraction, ensuring equal input volumes from individual specimens rather than normalization based on nucleic-acid concentration. This approach enabled broad virome characterization across a large number of field specimens while reducing the sequencing burden. However, pooled metagenomic sequencing limits the resolution of individual-level viral prevalence and direct associations between specific viruses and individual host characteristics or clinical status (Teufel and Sobetzko, 2022). In addition, nasal and anal swab specimens were combined before downstream processing, preventing assessment of anatomical compartment specificity. Consequently, the metagenomic data cannot determine whether individual viral taxa were predominantly associated with the respiratory or gastrointestinal tract, and their tissue distribution should therefore be interpreted cautiously. Moreover, because equal specimen volumes rather than equal nucleic-acid inputs were pooled, specimens with higher viral or nucleic-acid concentrations may have contributed disproportionately to the sequence abundance observed in individual pooled libraries. The seven analytical groups were also not mutually independent, because specimens included in the farming-system and seasonal comparisons were also represented within the corresponding geographic groups. Therefore, differences in viral contig abundance and community composition among pooled libraries should be interpreted as descriptive group-level patterns rather than independent estimates of ecological effects. Future studies using individual-level sequencing or independently replicated pools, together with more comprehensive host and farm metadata, will be needed to validate these associations and identify the factors underlying bovine virome variation.

An additional methodological limitation is the use of multiple displacement amplification (MDA) during DNA library preparation. MDA can introduce non-uniform amplification and preferentially amplify certain DNA templates, including circular DNA molecules, potentially affecting the relative representation of DNA viral taxa. In addition, dedicated extraction blanks, MDA negative controls, and library-preparation negative controls were not included in the original sequencing workflow, limiting our ability to formally exclude low-abundance reagent- or laboratory-associated signals. Accordingly, differences in DNA viral contig abundance should be interpreted as descriptive sequence-based patterns rather than quantitative estimates of biological abundance, and low-abundance DNA viral signals should be interpreted cautiously.

Phylogenetic analyses demonstrated that several viral sequences detected in cattle-derived specimens clustered with viruses previously identified in humans and other animal hosts, revealing genetic relationships among viruses associated with diverse vertebrate hosts. In particular, astroviruses, picobirnaviruses, and picornaviruses were distributed across multiple host-associated lineages, consistent with previous reports describing their broad host distributions. Although these phylogenetic relationships do not provide direct evidence of cross-species transmission, they highlight the potential for broader host associations and warrant further investigation of viral host range and host-switching at livestock–wildlife–human interfaces. Similar observations have been reported for circoviruses, with closely related circovirus sequences detected in different mammalian hosts, including cattle and pigs (Xu et al., 2022). More generally, the close genetic relationships observed among several viral groups identified in this study are consistent with previous reports of related viral lineages occurring across multiple host species (Roach and Langlois, 2021). Further host-resolved genomic and epidemiological studies will be required to determine whether these relationships reflect shared evolutionary ancestry or cross-species transmission events.

Animal movement and ecological connectivity represent plausible mechanisms that may contribute to viral dissemination among cattle populations. Frequent transportation associated with livestock trade can facilitate long-distance transmission of viral pathogens, whereas interactions with wildlife may provide additional opportunities for viral introduction into domestic animal populations. Consistent with this, bovine coronaviruses have been reported to spread rapidly among cattle populations and undergo genetic recombination, contributing to viral genetic diversity (Keha et al., 2019). Moreover, migratory wildlife has been implicated in the dissemination of multiple viral pathogens across geographic regions (McDuie et al., 2022). However, livestock movement, wildlife contact, and trade networks were not directly quantified in the present study; therefore, their contributions to the geographic and phylogenetic patterns observed here remain to be determined. Together, these factors highlight the complexity of viral transmission within interconnected livestock production systems and emphasize the importance of integrating virome surveillance with animal-movement and epidemiological data.

To provide targeted validation of selected metagenomic findings, we integrated targeted PCR/RT-PCR screening with cell-culture-based virus characterization, providing complementary validation of representative viral findings. Among the 293 cattle examined, bovine rotavirus (BRV), bovine norovirus (BNoV), and bovine coronavirus (BCoV) were the most frequently detected viruses, and multiple viral targets were detected in 71.92% of the cattle. The frequent co-detection of multiple viral targets indicates a co-detection pattern within the sampled cattle populations. However, detection of multiple viral nucleic acids does not necessarily demonstrate simultaneous active infection or biological interactions among the detected viruses. Previous studies have also reported frequent detection of multiple viral agents in cattle, particularly in animals with gastrointestinal disease (Su et al., 2022), although evidence of active co-infection depends on the diagnostic approaches used. Further studies incorporating individual specimen types, quantitative viral-load measurements, and detailed clinical information will be required to determine the biological and clinical significance of the co-detection patterns observed here.

Collectively, this study provides a large-scale characterization of the beef cattle virome in Northwest China, revealing extensive viral diversity and descriptive differences in viral community composition among the pooled libraries representing geographic regions, farming systems, and sampling seasons. Phylogenetic analyses revealed genetic relationships between several viral sequences detected in this study and viruses previously reported from diverse host species, highlighting hypotheses regarding broader host associations and potential cross-species transmission that require further investigation. Furthermore, the integration of viral metagenomics, targeted PCR/RT-PCR screening, and cell-culture-based virus characterization provided complementary validation of representative viral findings and revealed frequent co-detection of multiple viral targets within the sampled cattle populations. These findings expand current understanding of viral diversity and community patterns associated with beef cattle populations in Northwest China and establish a valuable resource for future studies of viral evolution, surveillance, and disease prevention.

## Data Availability

The datasets presented in this study can be found in online repositories. The names of the repository/repositories and accession number(s) can be found in the article/supplementary material.

## References

[ref1] Alocilla O. Monti G. (2022). Network analysis of cattle movements in Chile: implications for pathogen spread and control. Prev. Vet. Med. 204:105644. doi: 10.1016/j.prevetmed.2022.105644, 35429840

[ref2] Ambrose R. K. Blakebrough-Hall C. Gravel J. L. Gonzalez L. A. Mahony T. J. (2023). Characterisation of the upper respiratory tract Virome of feedlot cattle and its association with bovine respiratory disease. Viruses 15:455. doi: 10.3390/v15020455, 36851669 PMC9961997

[ref3] Bassi C. Guerriero P. Pierantoni M. Callegari E. Sabbioni S. (2022). Novel virus identification through metagenomics: a systematic review. Life 12:2048. doi: 10.3390/life12122048, 36556413 PMC9784588

[ref4] Hou X. Niu Z. Wu S. du Q. Liu G. Nie L. . (2025). Dairy cattle infection with bovine rotavirus at different growth stages and its impact on health and productivity. Animals (Basel) 15:1628. doi: 10.3390/ani15111628, 40509094 PMC12153802

[ref5] Keha A. Xue L. Yan S. Yue H. Tang C. (2019). Prevalence of a novel bovine coronavirus strain with a recombinant hemagglutinin/esterase gene in dairy calves in China. Transbound. Emerg. Dis. 66, 1971–1981. doi: 10.1111/tbed.13228, 31077561 PMC7168545

[ref6] Li C.-X. Shi M. Tian J.-H. Lin X.-D. Kang Y.-J. Chen L.-J. . (2015). Unprecedented genomic diversity of RNA viruses in arthropods reveals the ancestry of negative-sense RNA viruses. eLife 4:e05378. doi: 10.7554/eLife.05378, 25633976 PMC4384744

[ref7] McDuie F. Matchett E. L. Prosser D. J. Takekawa J. Y. Pitesky M. E. Lorenz A. A. . (2022). Pathways for avian influenza virus spread: GPS reveals wild waterfowl in commercial livestock facilities and connectivity with the natural wetland landscape. Transbound. Emerg. Dis. 69, 2898–2912. doi: 10.1111/tbed.14445, 34974641 PMC9788224

[ref8] Mokili J. L. Rohwer F. Dutilh B. E. (2012). Metagenomics and future perspectives in virus discovery. Curr. Opin. Virol. 2, 63–77. doi: 10.1016/j.coviro.2011.12.004, 22440968 PMC7102772

[ref9] Nantel-Fortier N. Gauthier M. L’Homme Y. Lachapelle V. Fravalo P. Brassard J. (2022). The swine enteric virome in a commercial production system and its association with neonatal diarrhea. Vet. Microbiol. 266:109366. doi: 10.1016/j.vetmic.2022.109366, 35176608

[ref10] Nelsen A. Knudsen D. Hause B. M. (2023). Identification of a novel Astrovirus associated with bovine respiratory disease. Transbound. Emerg. Dis. 2023, 1–8. doi: 10.1155/2023/8512021, 40303779 PMC12017199

[ref11] Nooij S. Schmitz D. Vennema H. Kroneman A. Koopmans M. P. G. (2018). Overview of virus metagenomic classification methods and their biological applications. Front. Microbiol. 9:749. doi: 10.3389/fmicb.2018.00749, 29740407 PMC5924777

[ref12] Park J. Kim E. B. (2020). Differences in microbiome and virome between cattle and horses in the same farm. Asian Australas. J. Anim. Sci. 33, 1042–1055. doi: 10.5713/ajas.19.0267, 32054207 PMC7206377

[ref13] Roach S. N. Langlois R. A. (2021). Intra- and cross-species transmission of Astroviruses. Viruses 13:1127. doi: 10.3390/v13061127, 34208242 PMC8230745

[ref14] Roper A. M. Orellana Rivas R. M. Gao J. Marins T. N. Savegnago C. G. Rodrigues T. . (2025). Seasonal effects on growth, digestibility, and metabolism of preweaning dairy calves in the southeastern United States. J. Dairy Sci. 108, 4318–4331. doi: 10.3168/jds.2024-25938, 39890068

[ref15] Shi M. Lin X. D. Chen X. Tian J. H. Chen L. J. Li K. . (2018). The evolutionary history of vertebrate RNA viruses. Nature 556, 197–202. doi: 10.1038/s41586-018-0012-7, 29618816

[ref16] Su N. . (2022). Prevalence of bovine viral diarrhea virus in cattle between 2010 and 2021: a global systematic review and meta-analysis. Front. Vet. Sci. 9:1086180. doi: 10.3389/fvets.2022.1086180 PMC9887317 36733426

[ref17] Teufel M. Sobetzko P. (2022). Reducing costs for DNA and RNA sequencing by sample pooling using a metagenomic approach. BMC Genomics 23:613. doi: 10.1186/s12864-022-08831-y, 35999507 PMC9400246

[ref18] Vlasova A. N. Saif L. J. (2021). Bovine coronavirus and the associated diseases. Front. Vet. Sci. 8:643220. doi: 10.3389/fvets.2021.643220, 33869323 PMC8044316

[ref19] Xia R. Chen Y. Yi P. Sun Y. Chen L. Ma X. . (2025). Fecal microbiota changes in Angus beef cows persistently infected by bovine viral diarrhea virus. Vet Sci 12:538. doi: 10.3390/vetsci12060538, 40559775 PMC12197573

[ref20] Xu T. Chen X. M. Fu Y. Ai Y. Wang D. M. Wei Z. Y. . (2022). Cross-species transmission of an emerging porcine circovirus (PCV4): first molecular detection and retrospective investigation in dairy cows. Vet. Microbiol. 273:109528. doi: 10.1016/j.vetmic.2022.109528, 35944390

[ref21] Yan M. Yu Z. (2024). Viruses contribute to microbial diversification in the rumen ecosystem and are associated with certain animal production traits. Microbiome 12:82. doi: 10.1186/s40168-024-01791-3, 38725064 PMC11080232

[ref22] Zhang S. Gao H. Zhang G. Fang M. Kong Y. Jiang L. . (2024). Metavirome analysis of domestic sheep in Shaanxi, Gansu, and Ningxia, China. Front. Vet. Sci. 11:1508617. doi: 10.3389/fvets.2024.1508617, 39691376 PMC11649628

[ref23] Zhang C. Hou L. (2020). Data middle platform construction: the strategy and practice of National Bureau of Statistics of China. Stat. J. IAOS 36, 979–986. doi: 10.3233/SJI-200754

