# Supplementary material for: Large-scale characterization of the bovine virome reveals viral diversity and community patterns in Northwest China
Source: Front Microbiol. 2026 Sep 22;17:1924807. doi: 10.3389/fmicb.2026.1924807 (PMC13638655; doi:10.3389/fmicb.2026.1924807)
Supplement: Supplementary file 1 [file Table_1.DOCX]

**Supplementary Data 1**

Original uncropped gel images corresponding to Figure 8A.

Images are presented without cropping or contrast enhancement, except for minimal adjustments applied uniformly across the entire image. All images correspond directly to the data shown in Figure 8A.

# Figure 8A-1. Original uncropped gel image.

# Figure 8A-2. Original uncropped gel image.

# Figure 8A-3. Original uncropped gel image.

# Figure 8A-4. Original uncropped gel image.

# Figure 8A-5. Original uncropped gel image.

# Figure 8A-6. Original uncropped gel image.
